# Suicide among users of mental health and addiction services in the first 10 months of the COVID-19 pandemic: observational study using national registry data

**DOI:** 10.1192/bjo.2022.510

**Published:** 2022-06-09

**Authors:** Fredrik A. Walby, Martin Ø. Myhre, Lars Mehlum

**Affiliations:** National Centre for Suicide Research and Prevention, Institute for Clinical Medicine, University of Oslo, Norway; National Centre for Suicide Research and Prevention, Institute for Clinical Medicine, University of Oslo, Norway; National Centre for Suicide Research and Prevention, Institute for Clinical Medicine, University of Oslo, Norway

**Keywords:** Suicide, mortality, in-patient treatment, out-patient treatment, epidemiology

## Abstract

Although many studies have reported no rise in suicides in the general population following the COVID-19 pandemic, little is known regarding mental health and substance misuse service patients, groups who have reportedly faced substantial reductions in their access to care during phases of lockdown. However, in this observational study using national registry data, during the first 10 months of the pandemic we found no evidence of an increased risk among people in recent (within 12 months) contact with secondary care. Both long-term and differential effects on subgroups remain to be studied.

The worldwide impact of the COVID-19 pandemic was predicted by many experts to lead to significant increases in world suicide rates,^1^ but this seems not to have happened, at least not during the first period of the pandemic.^2–4^ Still, there has been concern that vulnerable groups, such as people in need of treatment in mental health and addiction services,^5,6^ would be at increased risk of suicide. However, so far, no nationwide data on this group have been reported. In this brief report, based on nationwide registry data, we examine whether the national number of suicides among people in contact with mental health or addiction services increased during the first 10 months of the pandemic.

## Method

This study used a national registry linkage. First, we identified all suicides (ICD-10 codes X60–X84; Y10–Y34; Y87.0; Y87.2) in Norway between 1 January 2016 and 31 December 2020 from the Norwegian Cause of Death Registry (NCDR). Second, we linked all suicides from the NCDR with the Norwegian Patients Registry (NPR). The NPR contains data on contacts with secondary mental health services, secondary substance misuse services (which primarily treat alcohol and drug use disorders, with opioid use and use of multiple substances being most prevalent^7^), child and adolescent mental health services and private mental health specialists commissioned by a public health trust. Thereby, the linkage identified all people who died by suicide and had had contact with mental health or substance misuse services during the year before their death. From this linkage we aggregated the monthly number of suicides per year.

Data were analysed as an interrupted time series. We defined the time from 1 January 2016 to 28 February 2020 as the pre-pandemic period, since the first outbreak of COVID-19 and the resulting lockdown in Norway started in March. We used suicide counts from this period to forecast the expected number of suicides per month for the first 10 months of the pandemic (1 March 2020 to 31 December 2020) using Poisson regression. Fourier terms, which are pairs of sine and cosine functions, were used to control for seasonality. We compared the expected number of suicides with the observed number for the first 10 months of the pandemic and estimated rate ratios with 95% confidence intervals using the delta method. Data were analysed using R version 4.1.2 for Windows (www.r-project.org). We assert that all procedures contributing to this work comply with the ethical standards of the relevant national and institutional committees on human experimentation and with the Helsinki Declaration of 1975, as revised in 2008. All procedures involving patients were approved by The Norwegian Directorate of Health and the Regional Committees for Medical and Health Research Ethics, South-East Norway (approval no. 32494).

## Results

The overall prevalence of contact with mental health and substance misuse services among all people who died by suicide in Norway was 45.5% in 2020, compared with a mean yearly prevalence of 45.0% between 2016 and 2019. The mean monthly number of suicides among people who had contact with mental health and substance misuse services was identical in the pre-pandemic (24.8, s.d. = 4.83) and the post-pandemic period (24.8, s.d. = 6.99). In the Poisson model of the pre-pandemic period, we found that suicide numbers were increasing among people using mental health and substance misuse services in the pre-pandemic period (rate ratio 1.0001; 95% CI 1.0000–1.0002; *P* = 0.030). The model fit was good (*P* = 0.698).

As shown in Table 1, we observed an overall decrease in the number of suicides in the first 10 months of the pandemic, with a rate ratio of 0.87 (95% CI 0.73–1.03). All months except August and March had estimated ratios of observed and expected numbers below 1, but with overlapping confidence intervals. In absolute numbers this is equal to an observed reduction of 38 suicides (13%) during the period.

**Table 1:** Observed and expected number of suicides in people who had contact with mental health and substance misuse services during the first 10 months of the COVID-19 pandemic in 2020

	Suicides, *n*		
Month	Observed	Expected	Ratio (95% CI)
March	32	28.3	1.13 (0.68−1.87)
April	19	30.4	0.63 (0.35−1.11)
May	23	30.2	0.76 (0.44−1.31)
June	28	28.2	0.99 (0.59−1.68)
July	21	26.3	0.80 (0.45−1.41)
August	39	26.3	1.48 (0.90−2.43)
September	16	28.1	0.57 (0.31−1.05)
October	28	29.9	0.94 (0.56−1.57)
November	23	30.0	0.77 (0.45−1.32)
December	19	28.4	0.67 (0.38−1.20)
Total	248	286	0.87 (0.73−1.03)

## Discussion

We found no evidence of an increase of suicide in people who had contact with mental health and substance misuse services during the first 10 months of the COVID-19 pandemic in Norway. Rather, tendencies towards a reduction in the number of suicides in this group were observed in the context of no increase in suicide rates in the general population in Norway,^8^ where reduced rate ratios were found for March–May and October–December, and an increased rate ratio for June–September, all of which were non-significant differences well within the 95% prediction intervals. Additionally, no change in the overall prevalence of contact with services was found.

Our findings are well aligned with results from studies in the general population consistently finding no evidence of increased suicide mortality in the first period of the pandemic,^2^ although the long-term effects remain to be established. Also, whether this finding will be replicated in other countries and health systems is important to examine.

The universal health insurance of Norway and the comparatively high access to mental healthcare may, in part, help explain the observed absence of increases in suicides, in addition to the substantial governmental efforts that were implemented to mitigate the effects of the pandemic on society. Initial reductions in access to mental health and substance misuse services were of brief duration; great efforts were made by both governmental and other organisations to keep these services open and accessible, although research on actual performance of and possible disruptions to services are lacking. Increased use of video consultations was one of the main recommended compensatory actions. Some evidence shows that most general practitioners were technically able to provide this from early in the pandemic, and there is more preliminary data pointing in the same direction from mental health and substance misuse services.^9^

We were not able to examine possible differential effects on suicidal mortality among subgroups of people in contact with services before suicide. One important area to study is whether there are differences in the effect on suicide risk connected with the pandemic between, for example, people with major psychiatric disorders and those with more stress-induced conditions.

There is growing evidence of the large and widespread impact of the COVID-19 pandemic on societies worldwide, including adverse economic effects, increased mental distress^10^ and an increase in suicidal thoughts.^11^ The seeming lack of association with suicide rates at both the general population level and clinical level in a short time perspective merits considerable interest and might provide an opportunity for further study of risk and protective factors for suicide. Since long-term effects of the pandemic on suicide mortality remain to be established, it is necessary to stay vigilant both to possible effects on vulnerable groups, such as patients in contact with mental health and substance misuse services, as well as to possible changes in the prevalence of important risk factors in society in general.

## Data Availability

The data that support the findings of this study are available on GitHub (https://github.uio.no/martiom/suicide-covid19) or from the corresponding author on reasonable request.
